# Game theory and weight loss: Harmonic evidence from randomized controlled trials in Appalachia, KY and Jordan

**DOI:** 10.1371/journal.pgph.0004100

**Published:** 2025-02-07

**Authors:** Daniel E. Zoughbie, Dillon Huddleston, Eric L. Ding

**Affiliations:** 1 Department of Public Health, New England Complex Systems Institute, Cambridge, Massachusetts, United States of America; 2 Social Network Research Group, Microclinic International, San Francisco, California, United States of America; 3 Institute of International Studies, University of California at Berkeley, Berkeley, California, United States of America; Universiti Malaya, MALAYSIA

## Abstract

Both behavioral and experimental economics have a lengthy research history concerning the presence of and ability to support cooperation in infinitely repeated games. A workhorse model which has emerged for inferring players’ strategies from their actions in finitely repeated games, is applied to data from randomized controlled trials conducted in Jordan and Kentucky; an attractive economic application portending substantive public health and other policy consequences. We find that treatment significantly increases unconditional cooperation (from 27% of controls to 45% of treated subjects in Kentucky, and 38% to 85% in Jordan, with an intermediate level of 76% associated with partial treatment in the latter), and despite all attending cultural and other differences between these two geographies, treatment in each yielded significant and unimpeachable improvements in outcomes; improved program attendance and weight loss. Such harmonious results across vastly disparate geographies is highly unusual and correspondingly impressive. For the first time, we are showing increased cooperative behaviors causally associated with these trials.

## 1. Introduction

In (mathematical) economics, game theory is typically classified as being either competitive, in which players cannot form coalitions amongst themselves and compete with other coalitions, and cooperative in which case they can. The notion that one of these modes may transition into the other, via a hybrid tipping point referred to as cooperative competition or *coopetition* (Chiambaretto et al. 2019 [1]), however, is less established in economics. Alternatively, both the behavioural and experimental economic strands of literature have a long and growing history of research studying the presence of and ability to support cooperation in infinitely repeated, typically prisoner’s dilemma (PD), games which in practice are subdivided into sequences of *super-games*, each of these consisting of a sequence of stage games/periods for two fixed players. This literature was recently surveyed by Dal Bó and Fréchette 2018 [2]. A workhorse model that has emerged for inferring players’ strategies from their choices/actions in finitely repeated games is the *Strategy Frequency Estimation Method (SFEM)* of Dal Bó and Fréchette 2011 [3]; which estimates a mixture model weighted by the frequencies with which players follow each of a prespecified set of strategies (a popular benchmark with 20 common ones being given in Appendix H of Romero and Rosokha 2018 [4]). Players must be permitted to *tremble* (deviate from actions specified by their followed strategy), which in a two-action stage game such as the PD, implies that players act according to their followed strategy with some probability, *β* ∈ [0.5,1), and tremble otherwise (i.e., cooperate when the strategy demands defection, or vice versa). [Here, the tremble probability, *γ* ≡ 1 − *β*, is homogeneous/fixed across players/subjects within treatment groups, though Bland 2020 [5] has since extended the methodology to allow for heterogeneity of such subjects’ tremble probabilities.] Maximum likelihood estimates (MLEs) may then be obtained for *β* and the mixing frequencies over the set of (*K*) prespecified strategies(∀*k* ∈ {1*,...,K*}) *ϕ*_*k*_ ∈ [0,1]: ∑*ϕ*_*k*_ = 1.

Such an implementation of the SFEM due to Romero and Rosokha 2018 [4] is separately applied to clinical, survey and social network data from randomized controlled trials (RCTs) conducted in Amman, Jordan and Appalachian Bell County, Kentucky. Qualitatively similar research questions have been addressed through RCTs by Van Ryzin, Roseth and Biglan 2020 [6], who find that cooperative learning significantly boosts students’ prosocial behaviours. We hypothesize that the Microclinic Social Network Program (MSNP), which was originally designed as a game to test the possibilities and limitations of what we term “Managed Co-opetition,” and which is structured analogously to cooperative learning, will improve cooperative behaviors and weight loss.

## 2. Methods

Following the notation and general procedure of Romero and Rosokha 2018 [4], given the (*N*) subjects, the SFEM begins with two definitions:


$$\left(\forall n\;\in \;\{1,...,N\}\right)\left(\forall k\;\in \;\{1,...,K\}\right)\;C\left(k,n\right)\;\equiv \;\left|\left\{n\;plays\;k\right\}\right|\;and\;E\left(k,n\right)\;\equiv \;\left|\left\{\neg \left(n\;plays\;k\right)\right\}\right|$$

i.e., *C* (*E*) is a *N* × *K* matrix recording the number of periods during which player *n* does (not) play according to strategy *k*; meaning that player does (not) cooperate when the strategy demands cooperation and likewise for defection. The following Hadamard (element-wise) *N* × *K* matrix product is then defined (and used for element-wise exponentiation also):


$$\left(\forall n\;\in \;\left\{1,...,N\right\}\right)\left(\forall k\;\in \;\left\{1,...,K\right\}\right)\;P\left(k,n\right)\;\equiv \;\beta^{{{}^{C}}^{\left(k,n\right)}}{\left(1\;-\;\beta \right)}^{{{}^{E}}^{\left(k,n\right)}}$$

This is compactly denoted *P* ≡ *β*^*C*^ ⊙ (1 − *β*)^*E*^ = *β*^*C*^ ⊙ *γ*^*E*^ using the (homogeneous) trembling probability. As such, *P* records the (binomial) likelihood that the sequential actions of subject *n* are generated by strategy *k*. Denoting by *ϕ* (*ι*) a column (row) *K*(*N*)-vector of mixing frequencies (ones), the log-likelihood follows:


$$l\left(\beta ,\phi \right)\;\equiv \;\iota ln\left(P\phi \right)$$

The SFEM then proceeds by maximizing this expression over (*β,ϕ*) ∈ [0.5,1)×[0,1]^*K*^, subject to the constraint ∑*ϕ*_*k*_ = 1; pseudorandomly initializing many (5000) times to avoid local maxima.

As the RCT data presently considered were not generated from an experimental design (computer-delivered or otherwise) dedicated to (or even at the time necessarily envisioning the eventual use of) the repeated PD, the question of how to define cooperation vs. defection is less straightforward: In line with the goals of the MSNP, we interpret cooperation in the sense of commitment; i.e., that subjects commit to attend the program/relevant treatment and follow its instructions in between sessions. If this has been done (meaning subjects have strictly adhered to dietary and exercise requirements), then the subject weight should not increase; thus if a given subject attends a given session and has a weight not exceeding that at the previous session (also attended), then the subject is taken to have cooperated; otherwise they have defected. This allows direct cooperation/defection determination from the RCT weight and attendance data in the results that follow. Other mappings from the RCT data into cooperation/defection decision determination could be considered, but we consider the chosen one to be in keeping with both the understanding of the PD as a conflict between commitment and temptation to defect and the weight loss goal of the administered treatment: The temptation to defect may be seen as short-term thinking (the immediate satisfaction of consuming unhealthy foods or not exercising, e.g.) versus commitment to long-term health improvement. The socially networked nature of the MSNP hypothesizes that grouping (and in the subsequent analysis, in particular, partnering) of treatment subjects generates a ‘buddy’ effect (Feltz, Kerr and Irwin 2011 [7]) whereby individuals support their own improvement by committing to undertake the hardship of the program in concert with their partner(s), anticipating that it is more difficult to abandon one’s ‘team’ than to simply individually relent. This is an instance of the more general Köhler effect (Kerr et al. 2000 [8]) motivating subjects to keep up with their group members in order to avoid falling behind and being a weak link.

As noted, the RCT data are restricted to doubleton microclusters, which other than individuals are most common in both geographies; and given that treatment randomization was independent of microcluster size, should in no way disturb causal inference. This is done as the most natural embedding of the RCT data (via the chosen cooperation/defection mapping) into the experimental repeated PD literature, which is mostly concentrated on two-player games. (Of course, this is the case for the PD itself, and all 20 canonical strategies in Appendix H of Romero and Rosokha 2018 [4]. Another advantage of the RCT data is that subjects play one another, so each stage game generates a cooperation/defection decision for two subjects, whereas this is only one for the majority of published experiments in which subjects play computerized partners.) It was attempted to decompose microclusters with more than two members into all unique pairs, and otherwise proceed the same; gradually yielding less sharp results as larger microclusters were incorporated: It is hypothesized that this may be due to ‘strategy dilution’ whereby such subjects play the same strategy/sequence of cooperation/defection choices against more than one opponent, regardless of the opponent’s play; meaning the MLE will be less effectively able to match observed play with the 20 canonical strategies, and in particular, that a higher trembling probability will result. Furthermore, the pairwise decomposition will accord a much greater presence in the RCT data to larger microclusters and the strategies their members play; possibly also impacting the treatment randomization and causal inference. Finally, an additional, intuitive explanation may be that there is reduced bonding in larger microclusters, and as such lesser pairwise ‘buddy’ effects.

The Python SFEM implementation of Romero and Rosokha 2018 [4] was expediently applied to the present RCT data, but note also that an R framework has been developed by Dvorak 2023 [9]. Among the wealth of literature and methodology for inferring subjects’ strategies from their choices in repeated games reviewed by Dal Bó and Fréchette 2018 [2], the one-period ahead strategy method of Vespa (2017) [10] is notable as a viable alternative to analyzing the RCT data, but again, the SFEM was chosen as it represents more of a ‘gold standard’ workhorse model within this literature. Finally, a very recent and apparently general nonparametric method due to Aradillas-López and Kosenkova (2023) [11] may also eventually be usefully applied to the RCT data, but similarly, simply has not had time to become established in the literature and potentially displace the SFEM as the go-to methodology for strategy inference in repeated games.

As discussed, the PD is both the canonical stage game studied in the experimental repeated games literature, as well as one which intuitively fits into the present RCT context as an individual struggle between attending and observing program requirements (and as such losing weight) in honouring the commitment made to one’s microcluster by cooperating; versus defecting by succumbing to the short-term temptation to skip program sessions or engage in unhealthy behaviours (poor diet or lack of exercise) in between. Nonetheless, the experimental repeated games literature has concerned itself with several other stage games, as reviewed by Dal Bó and Fréchette 2018 [2]: Coordination games (Cooper 1999 [12]) reward players acting in concert (i.e., coordinating); which could sensibly apply to subject cooperation improving each individual’s outcome, but in the long term, joint defection will harm all individuals the most. Trust (an elaboration of the dictator) games (Berg, Dickhaut and McCabe 1995 [13]) capture interesting but asymmetric relationships that are apparently not suited to the present RCT data, assuming that microcluster members are better represented as (equal) partners than trustor/trustee in some sense. This is similarly the case for gift-exchange games (Akerlof and Yellen 1990 [14]) and employer/employee relationships. Partnership (a particular symmetric) games (Robinson and Goforth 2005 [15]) partly capture the notion of interdependence among subject outcomes, but fully rather than partially; their assumption that a cooperator and defector see the same result regardless of roles is clearly inapplicable to weight loss. Public goods games have a long history in experimental economics (Fehr and Gaechter 2002 [16]) but their notion that the payoff (public good) is evenly divided amongst players is similarly inapplicable; one’s weight loss is one’s own. Thus aside from being the canonical stage game in the experimental repeated games literature, the PD may also be the most naturally fitting for the present RCT data (notwithstanding less notable stage games discussed by Dal Bó and Fréchette 2018 [2] or otherwise). That said, the SFEM is agnostic of the stage game, though the 20 strategies from Appendix H of Romero and Rosokha 2018 [4] are associated with the PD.

### 2.1. Intervention

The MSNP was designed as a gamified, win-win public health program with the aim of facilitating “Managed Coopetition.” That is to say forces of social cooperation and competition were optimized by a trained facilitator towards the primary goal of weight loss. In the RCT treatment group, subjects were partitioned into micro-clusters (“microclinics”) which effectively constitute coalitions jointly completing the program: within classroom and field settings (e.g., grocery stores for dietary lessons or the gym for those in exercise), instruction was provided to a class consisting of several disjoint (micro)clusters, each typically including two or three subjects. Significant individual progress toward weight loss was rewarded with achievement recognition each session, fostering competition both within and between clusters.

Whereas it was hypothesized that cooperation within and between clusters could arise as subjects (a) develop additional social ties/interdependencies, (b) are motivated by the progress of others, and (c) explicitly or implicitly recognize that in order to achieve individual progress, it behooves them to “train” in coordination with their own cluster; a sort of rivalry or “friendly,” cooperative competition which has been termed coopetition emerges. This coopetition was hypothesized to create towards to top, i.e., a weight loss cascade. The full trial methods, including consort diagrams, have been documented elsewhere (Zoughbie et al 2023 [17], Ding et al 2024 [18]).

Collected in Table 1 are the baseline characteristics of the trial participants in Kentucky.

**Table 1:** Baseline characteristics of Kentucky trial participants.

Characteristic, mean ± SD, or %	Intervention Group (n = 301)	Control Group (n = 193)
Age, years	52.7 ± 13.7	56.6 ± 13.3
Women, (%)	88.4	84.1
Weight, kg	94.7 ± 20.0	95.7 ± 21.6
Height, m	1.63±	1.64±
BMI, kg/m^2^	36.2 ± 7.4	36.0 ± 8.0
Obesity, BMI>=30, %	78.2	76.5
Waist circumference, inches	44.1 ± 5.5	44.5 ± 5.7
HbA1c %	6.1	6.2
HDL cholesterol, mg/dl	42.3 ± 13.2	38.9 ± 12.2
Mean arterial blood pressure, mmHg	95.6 ± 10.4	96.0 ± 10.2

Collected in Table 2 are the baseline characteristics of the trial participants in Jordan.

**Table 2:** Baseline characteristics of Jordan trial participants.

Characteristic, mean (SD) or %	Full MCP (n = 537)	Basic MCP (n = 186)	Control Group (n = 187)
Age, years	54.2	56.6	56.2
Women, (%)	66.5	67.0	65.0
Weight, kg	85.9	85.0	86.0
Height, m	160.3	159.6	160.3
BMI, kg/m^2^	33.6	33.5	33.4
Systolic blood pressure, mm Hg	129.1	131.7	132.2
Diastolic blood pressure, mm Hg	81.3	81.0	81.3
Mean arterial pressure, mm Hg	97.2	97.9	98.3
HbA1c (%) (SD)	6.91	6.90	6.91
Fasting plasma glucose*, mg/dL	147.8	142.8	145.7

* baseline fasting glucose average of first and second weeks.

## 3. Results

The results for each geography are first presented separately and then discussed together. Note that the interval between two sessions is one week in each geography. Whilst weight is independently considered the quintessential endpoint in public health obesity RCT studies, it can be meaningfully gained/lost on this one-week time scale, whereas alternate endpoints like fasting blood sugar vary on a much more rapid scale (sugar can spike in minutes) and are subject to the vagaries of daily patient food intake and activity. Others like glycated hemoglobin vary on much less rapid scales (it takes three months) and are unlikely to contemporaneously respond to weekly program participation.

### 3.1. Jordan

Collected in Table 3 are the SFEM MLEs obtained for Jordan. The rows correspond to full (A) versus partial (B) treatment versus control (C).

**Table 3:** Jordan SFEM MLEs.

Treatment	*β*	*ϕallc*	*ϕctod*	*ϕtft*	*ϕ*2*tf*2*t*	*ϕalld*	−*ℓ*
A	0.65	0.85	0.14	0	0	0	1030
B	0.68	0.76	0	0	0	0.15	558
C	0.67	0.38	0	0.35	0.26	0	269

Note that only strategies for which at least one group’s members in at least one geography are estimated to play with at least 10% frequency are presented, to avoid discussing (much) less relevant and more complicated strategies; those above are collected in Table 4 (from Appendix H of Romero and Rosokha 2018 [4] and first proposed in Table 2 of Fudenberg, Rand and Draber 2012 [19]).

**Table 4:** Strategies exceeding 10% estimated frequency in one geography.

Abbreviation	Strategy
allc	Always cooperate
ctod	Cooperate then always defect
tft	Tit-for-tat; cooperate than emulate opponent
2tf2t	Two-tit-for-two-tat; cooperate than emulate opponent (single) *repetition*
alld	Always defect

Note that *all MLEs may be regarded as exact* since care was taken to avoid local maxima and there is otherwise no statistical uncertainty inherent in the log-likelihood function. (Though there could be some measurement error, e.g., in the weight and attendance taken to specify periodic cooperation or defection; and perhaps more robustly, global optimization could be considered in order to avoid pseudorandomizing several parameter initializations.) Thus the obtained values for *ϕ*_*allc*_ yield a stunning conclusion in Jordan; that *corresponding to full (A) versus partial (B) treatment versus control (C), there are respectively always-cooperation percentages of* 85 *>* 76 *>* 38*!*  i.e., unconditional program attendance and observance *doubles* in response to partial treatment versus control and increases more than a further 10% in response to full treatment. Furthermore, trembling rates are comparatively consistent across treatment groups; 35 vs. 32 vs. 33 percent in full vs. partial treatment vs. control. As such, this result indeed indicates an unequivocal, substantially increased (but comparatively no more or less consistent) rate of program attendance and observance (weight loss) in successive responses to partial and full treatment. The other MLEs are less impactful: Between 14–15% of the treatment groups exhibit (near-) always-defecting behaviour; and the balance 61% of controls display tit-for-tat behaviours, likely artifacts of the lack of coordination between microcluster partners in the control group.

### 3.2. Kentucky

Collected in Table 5 are the SFEM MLEs obtained for Kentucky. The rows correspond to treatment (A) versus control (C).

**Table 5:** Kentucky SFEM MLEs.

Treatment	*β*	*ϕallc*	*ϕctod*	*ϕtft*	*ϕ*2*tf*2*t*	*ϕalld*	−*ℓ*
A	0.84	0.45	0.28	0.01	0	0.01	176
C	0.89	0.27	0.57	0.05	0.02	0	168

As in Jordan, the obtained values for *ϕ*_*allc*_ yield an impactful conclusion in Kentucky; that *corresponding to treatment (A) versus control (C), there are alwayscooperation percentages of* 45 *>* 27*!* i.e., unconditional program attendance and observance *increases by two-thirds* in response to treatment versus control. Furthermore, trembling rates are comparatively consistent across treatment groups; 16 vs. 11 percent in treatment vs. control. As such, this result indeed indicates an unequivocal, substantially increased (but comparatively no more or less consistent) rate of program attendance and observance (weight loss) in response to treatment. The other MLEs are less impactful: Primarily of note is that similar but reversed proportions, 28% of the treatment and 57% of the control group, exhibit cooperate-then-always-defecting behaviour; likely artifacts of dropout following first sessions.

### 3.3. Both geographies

Perhaps the most resounding between the results for Jordan and Kentucky is their ‘rhythm:’ Despite all attending cultural and other differences between these two geographies, treatment in each yielded significant and unimpeachable improvements in outcomes; improved program attendance and weight loss. Of less note is that there is an apparent inverse relationship between always-cooperation levels and consistency: Lower levels of the latter in Jordan (65–68%) vs. Kentucky (84–89%) correspond to higher ones of the former (38 *<* 76 *<* 85 vs. 27 *<* 45). Whether this is evidence of a deeper relationship (that higher levels of program commitment are implemented less consistently) will require that further geographies be assessed, and in any case is tangential and of second-order compared to the present results.

To the best of our knowledge, this is the first study applying a modern, experimental economics technique for inference in repeated games such as the SFEM, to real-world public health RCT data such as that obtained from the MSNP in Jordan and Kentucky. Thus there is no possibility for direct comparison with prior studies, but concerning the experimental economics (as opposed to public health RCT) side, our study has both strengths and weaknesses: Namely, the primarily computer-based experiments generating data for such studies have the comparative strength of easily generating an abundance of data in the form of super-games amenable to more exacting statistical analysis; whereas our RCT data has the comparative strength of being ‘real-world’ and otherwise meaningful (for public health) in a manner that other studies cannot claim. The statistically significant results that we were able to obtain with the limited RCT data, however, are very strong even in the context of ‘typical’ computer-based experimental economics studies, e.g., as surveyed by Dal Bó and Fréchette 2018 [2].

## 4. Discussion

We have demonstrated in this paper that different variations of the Microclinic Social Network Program induced both substantial increases in cooperative behavior and weight loss. Because the trial was randomized and weight loss was pre-specified as an endpoint in both geographies, we are able to disentangle homophily and confounding (genetic, environmental, etc.) from causation. This is a very attractive, real-world medical/epidemiological application of the SFEM and behavioural/experimental economics more generally, portending substantive public (health) and other policy consequences. A core indication of program efficacy is developed, and in the spirit of the behavioural and experimental economics literature on cooperation in repeated games, it is determined how subjects *do* rather than how they (optimally) *should* behave. In particular, program participation and commitment is not strategic at all, and becomes less so the greater the degree of treatment, which in turn yields greater health improvements.

The significance of these findings should not be understood apart from their field context. The literature surveyed by Dal Bó and Fréchette 2018 [2] typically relies on computer-based experiments in which subjects (usually graduate students or other academic staff) play against computer programs which simulate pre-specified strategies (possibly with trembling). That we were able to perform this analysis on data sourced from two different trials in two vastly different socio-economic, cultural, and political settings is noteworthy. For starters, Jordan is highly urbanized and predominantly Arab; observance of the Islamic faith is high. Appalachia is rural and predominately White; observance of the Christian faith is high. Interestingly, economic deprivation and a high incidence of noncommunicable diseases are common denominators, perhaps suggesting that low-income communities may be particularly well-suited for interventions designed to promote cooperation.

Perhaps the most resounding quality between the results for Jordan and Kentucky is their ‘harmony.’ Despite all attending differences between these two contexts, treatment in each yielded significant and unimpeachable improvements in outcomes; improved program attendance and weight loss. Of less note is that there is an apparent inverse relationship between always-cooperation levels and consistency: Lower levels of the latter in Jordan (65–68%) vs. Kentucky (84–89%) correspond to higher ones of the former (38 *<* 76 *<* 85 vs. 27 *<* 45). Whether this is evidence of a deeper relationship (that higher levels of program commitment are implemented less consistently) will require that further geographies be assessed, and in any case is tangential and of second-order compared to the present results.

Finally, because both trials were implemented with government cooperation (the Bell County Department of Public Health in Kentucky and the Jordanian Ministry of Health and Queen Rania’s Royal Health Awareness Society), a major finding of this analysis is that governments can improve micro-level cooperation in ways that improve macro-level public health outcomes. MSNP is one concrete example of how to do this; one could imagine many other public health and societal challenges that could similarly be addressed through social network interventions. This suggests that additional research is needed to investigate how governments can not only improve public health outcomes, but as importantly, leverage social networks to induce cascading health improvements through improved cooperation. Results presented here are also consistent with observational and randomized trials showing improvements in other chronic disease management programs that utilized the Microclinic social network model in the following different geographical and socio-economic contexts: Jordan, Lebanon, Palestine, and Kentucky (Zoughbie et al. 2014 [20]; Zoughbie et al. 2009 [21]; Shahin et al. 2018 [22]; Ding et al. 2013 [23]; Prescott et al. 2013 [24]; Ding et al. 2013 [25]; Zoughbie et al. 2024 [17]; Zoughbie, Huddleston and Ding in press [26]; Ding et al. 2024 [18]). Now, for the first time, we are showing increased cooperative behaviors were causally associated with the MSNP.

This study was not without limitations. The RCT data is comparatively deficient in that each geography can only be construed as a single super-game for each subject, rather than tens as is common in computer experiments, each consisting of no more (and often much less) than 13 repeated stage games, versus many more in computer experiment stage games. And of course, the RCTs were not explicitly designed as repeated games, PD or otherwise, to test subject propensity toward cooperation vs. defection/competition; though the MSNP was initially designed as a game to test the possibilities and limitations of what we term “Managed Co-opetition.”

As such, relatively weak results might be expected from the RCT data. However, we conversely find very strong evidence that (unconditional) cooperation increases significantly from control to partial (in Jordan) to full treatment. This indicates that (partial) treatment (to a lesser degree) substantially increases subject program commitment, defined in terms of attendance and weekly weight loss, relative to control and independently of other (within-microcluster) subjects’ actions. Of course, Kentucky having been a two- rather than three-arm trial as in Jordan, the conclusions concerning partial treatment apply only to the latter.
